# Presentation and temporal nature of postacute sequelae of SARS-CoV-2 infection in a US national cohort

**DOI:** 10.1016/j.bbih.2026.101205

**Published:** 2026-02-27

**Authors:** Melinda L. Jackson, Matthew D. Weaver, Prerna Varma, Mark É. Czeisler, Lauren A. Booker, Christine F. McDonald, Rebecca Robbins, Anna Ridgers, Rashon Lane, Shantha M.W. Rajaratnam, Charles A. Czeisler, Stuart F. Quan, Mark E. Howard

**Affiliations:** a Turner Institute for Brain and Mental Health, School of Psychological Sciences, Monash University, Melbourne, Victoria, Australia; b Institute for Breathing and Sleep, Austin Health, Heidelberg, Victoria, Australia; c Department of Medicine, Mass General Brigham, Boston, MA, USA; d Division of Sleep Medicine, Harvard Medical School, Boston, MA, USA; e Violet Vines Marshman Centre for Rural Health Research, La Trobe Rural Health School, La Trobe University, Bendigo, Victoria, Australia; f Department of Medicine, The University of Melbourne, Melbourne, VIC, Australia; g Department of Respiratory and Sleep Medicine, Austin Health, Heidelberg, VIC, Australia

**Keywords:** COVID-19, Cluster analysis, Long COVID, Cognition, Mental health, Physical symptoms

## Abstract

**Background:**

Postacute Sequelae of SARS-CoV-2 Infection (PASC) is a novel illness emerging from SARS-CoV-2 infection, characterized by the persistence of symptoms beyond resolution of the initial illness. Understanding the phenotypes and trajectory of PASC is critical for diagnosis and development of personalized management approaches for specific PASC syndromes, to ultimately improve quality of life. The aim of this study was to examine the prevalence and duration, and characterize patterns of specific PASC symptoms in a community-based cohort.

**Method:**

A non-probabilistic, weighted, cross-sectional survey of 14,964 adults from the US general population from the COVID-19 Outbreak Public Evaluation (COPE) Initiative between March 10, 2022 and June 2, 2022.

**Results:**

At 1-month post-infection, 44% of individuals with a history of SARS-CoV-2 infection reported at least one physical symptom, 47% reported at least one mental health symptom and 33% reported at least one cognitive symptom (14%, 10%, and 17% in controls, respectively). The prevalence of specific physical, cognitive and mental health symptoms declined between 1- and 12-months post-infection, to a prevalence rate similar to non-infected controls by 12 months. Cluster analysis revealed the persistence of symptom groups, with loss of taste and smell, psychosocial symptoms, respiratory and cardiovascular symptoms, and brain fog the most prominent at 12 months. Although cognitive symptom clusters persisted to 6 months post-infection, mental health clusters were transient.

**Conclusions:**

The prevalence of most common PASC symptoms fell to general population levels within 12 months. Persisting physical and cognitive symptom clusters provide insights into potentially distinct disease pathways, and are essential for guiding clinical management strategies, rehabilitation programs, and public health interventions aimed at mitigating long-term impacts of COVID-19.

## Highlights

• In a large population cohort of US adults, PASC symptoms persist in clusters, with distinct patterns.
• Loss of taste and smell remained elevated in cases vs controls at 12 months.
• Cognitive and mental health symptoms lasted 6-12 months in cases.
• Physical, cognitive and mental health symptoms at 12 months were similar to controls.
• PASC symptoms were more frequent in cases vs controls.

## 1 Introduction

Post-acute sequelae of COVID-19 (PASC), or “long COVID”, is an illness emerging from SARS-CoV-2 infection, characterized by the persistence or development of symptoms beyond the initial illness or diagnosis, typically lasting for more than four weeks. PASC is a major global public health concern, with prevalence estimates ranging from 7 to 23% of individuals who have had a SARS-CoV-2 infection, depending on how it is defined (Carfì et al., 2020; Thaweethai et al., 2023; Ely et al., 2024). Significant social and economic impacts (Carfì et al., 2020) include occupational impacts for ∼4 million people in the US due to long-COVID symptoms, resulting in $US170–230 billion in lost wages annually (Pinto et al., 2022). PASC is a heterogeneous entity with diverse clinical manifestations, encompassing a wide range of symptoms affecting multiple organ systems, which can persist for weeks to months after the acute phase of COVID-19 (Lambert et al., 2024).

While PASC generally manifests within 1-3 months post infection, its presentation and symptomology are wide-ranging (Ahrnsbrak and Fang, 2024). This variability relates partly to inconsistencies in how PASC is defined, ranging from persistent to new or continuing symptoms, between 1 and 3 months duration, and variable symptom requirements (Thaweethai et al., 2023; National Institute for Health and Care Excellence, 2020). The heterogeneity of patient populations, overlapping symptoms and limited inclusion of control groups make it harder to characterize the trajectory of PASC symptoms. In a systematic review of PASC in previously hospitalized patients, fatigue, shortness of breath and joint pain were the most common symptoms, with 75% of individuals endorsing at symptoms at 8-12 months (Kelly et al., 2023). Conversely, in a systematic review of 76 studies across general and clinical populations, apart from fatigue and shortness of breath, sleep disorders, post-exertional malaise and anxiety were the most prevalent symptoms reported 1-3 months post SARS-CoV-2 infection (Kuodi et al., 2023). While symptoms decline substantially after six months, over 20% of patients continue to report post-exertional malaise, depression and anxiety. The few studies including comparison with a control population found that infected patients reported greater symptoms than population controls at 3, 6 and 12 months post-infection (de Bruijn et al., 2025; Huynh et al., 2023), however the prevalence was only significantly higher than test-negative controls at 3 months (de Bruijn et al., 2025). Collectively, these findings highlight the complexity of PASC as a multisystem condition with a wide spectrum of symptoms. While the frequency of most symptoms tends to decrease over 3-6 months, symptoms may persist or even increase in some individuals, underscoring the need for further characterizing this condition, particularly through comparison with uninfected control populations.

Clustering can be a valuable approach to identify pathological mechanisms and to inform targeted interventions based on distinct clinical phenotypes (Kuodi et al., 2023; Frontera et al., 2022; Fernández-de-las-Peñas et al., 2023). In a meta-analysis of PASC syndromes (Kuodi et al., 2023), seven studies were clustered to identify symptom profiles, from which three themes emerged: 1) cardiorespiratory (pooled estimate 36% [32-40%]), 2) systemic inflammatory (46% [17-77%]) and 3) neurological (72% [45-92%]). Other research using factor analysis identified 5 factors: cold and flu-like symptoms, change in smell and/or taste, dyspnea and chest pain, cognitive and visual problems, and cardiac symptoms (Pinto et al., 2022). This supports the emerging evidence that PASC occurs in clinical sub-types.

We used a nationally representative sample of US adults from the COVID-19 Outbreak Public Evaluation (COPE) Initiative (www.thecopeinitiative.org) to identify: 1) the prevalence of specific PASC symptoms over 12 months in affected individuals compared to individuals who reported no prior infection; and 2) significant clusters of PASC symptoms by time since reported infection.

## 2 Methods

### 2.1 Study design and participants

The COPE Initiative undertook a series of non-probabilistic, weighted, internet-based surveys. Cross-sectional analyses were undertaken for the prevalence and sequelae of SARS-CoV-2infection in the general population between March 10, 2022 and June 2, 2022. Three successive surveys were administered: Wave 1 (March 10-30, 2022), Wave 2 (April 4-May 1, 2022) and Wave 3 (May 4-June 2, 2022). Recruitment details have been described previously (Czeisler et al., 2021, 2024). Invitations were received by 27,932 eligible participants and 15,057 completed the survey (response rate 54%). Monash University Human Research Ethics Committee approved the study (#24036). Participants provided informed consent prior to initiating the survey.

### 2.2 Survey items and classification

Respondents were asked demographic questions and self-reported past and current medical conditions from a provided list, including diabetes, sleep disorders, cancer, cardiovascular disease and hypertension. , R espondents were then asked a series of questions to ascertain prior COVID-19 infection (Quan et al., 2023; Quan Stuart et al., 2024). Using branching logic with an initiating sequence of “Have you ever been tested for COVID-19?”, respondents who answered “Yes” were asked, “Have you ever tested positive?”. Those who denied prior COVID-19 testing were asked, “Despite never testing positive, are you confident that you have had COVID-19?” and “Despite never testing positive, have you received a clinical diagnosis of COVID19?”. Number of infections and the estimated date of each infection were collected. All participants were further asked “Have you experienced a problem with decreased sense of smell or taste at any point since January 2020?” (Hopkins et al., 2009). A positive history of SARS-CoV-2 infection was defined as an affirmative response to having tested positive for SARS-CoV-2, a clinical diagnosis of SARS-CoV-2, or loss of taste or smell (Callejón-Leblic et al., 2022; Gerkin et al., 2021). Those who responded “No” to testing positive or experiencing a loss of sense of taste or smell were considered controls. In the case of a positive SAR-CoV2, infection severity was assessed using self-reported indicators based on WHO Definitions, including whether the respondent had been hospitalized, required supplemental oxygen or was admitted to an intensive care unit (ICU) during their illness (World Health Organization, 2020).

Questionnaires characterizing symptoms that persisted >2 weeks after SARS-CoV-2 infection were administered, to ascertain the presence/absence and duration of common symptoms (Quan et al., 2023). For each symptom, participants who endorsed having experienced the symptom >2 weeks after their SARS-CoV-2 infection were asked how long their symptoms persisted after infection onset. Symptom duration was collected as a categorical variable capped at “Greater than 12 months” for physical and mood questionnaires, and “More than 6 months” for cognitive symptoms. Participants could also respond that they were still presently experiencing the symptom. Symptom questionnaires were completed separately for each infection. Symptom lists included respiratory, constitutional, neurological, cognitive and physical activity, informed by available evidence describing enduring symptoms following SARS-CoV-2 infection (Groff et al., 2021) and clinical assessment tools, such as the top-ranked “brain fog” descriptors in patients with postural tachycardia syndrome (Ross et al., 2013) (see Supplementary file for symptom list) Mood symptoms were derived from specific items of the Patient Health Questionnaire 4 (PHQ-4), using clinical cut-offs of 3 or greater on the Depression or Anxiety subscale (Kroenke et al., 2009). The PHQ-4 demonstrated excellent internal consistency (α = 0.92).

PASC was defined as the presence of at least one enduring physical, cognitive, or mood symptom for >4 weeks (Thaweethai et al., 2023; National Institute for Health and Care Excellence, 2020). Participants with no history of SARS-CoV-2 infection were considered test-negative controls. In Wave 1, controls were administered the same physical symptom assessment as cases and asked to reference the previous 2 weeks in reporting any of the listed symptoms. Controls were presented with all symptom assessments completed by cases in Waves 2 and 3, with directions to report the presence/absence of each listed symptom in the previous 2 weeks.

### 2.3 Data analyses

Demographic characteristics of the overall sample are presented as frequencies and percentages. For all analyses, alpha was set at <0.05. Differences in demographic characteristics by infection and enduring symptom status were compared using ANOVA or the Kruskal Wallis test.

We describe the prevalence of physical, cognitive, and mood symptoms among those who met the definition of PASC for time periods from more than 1 to over 12 months post infection, and those characterized as controls. Principal Factor Analysis (PFA) was implemented with the intent of identifying clusters of symptoms that may represent distinct PASC phenotypes. Factor loadings were rotated using the Varimax method. Items with eigenvalues <0.4 or factor loadings <0.3 were omitted. Correlation matrices between identified factors were inspected. Items loading with sufficient strength to multiple factors were assigned to the cluster with the strongest estimated loading factor. All analyses were conducted using Stata/SE 15.1 (StataCorp LLC, College Station, TX).

## 3 Results

Characteristics of the 14,964 participants in the sample are displayed in eTable 1 in the Supplement. Table 1 displays demographic and comorbid medical conditions stratified by PASC status and duration. Of those who endorsed having a prior SARS-CoV-2 infection (n = 5807 [38.8%]), symptoms of PASC were reported for ≥1 & <3 months by 10% of the sample, and ≥3 & <6 months by 14% of the sample. PASC symptoms persisted ≥6 months for 34% of the sample. Compared to those with no PASC, respondents with PASC symptoms >6 months endorsed significantly more diagnosed medical conditions, comorbidities and greater infection severity (Table 1).

**Table 1:** Characteristics of individuals with and without a history of SARS-CoV-2 infection stratified by PASC status and duration.

	Control No Positive Tests or Symptoms 61% (n = 9157)	Infection No PASC 16% (n = 2442)	Infection PASC (≥1 & <3 months) 10% (n = 577)	Infection PASC (≥3 & <6 months) 14% (n = 801)	Infection PASC (≥6 months) 34% (n = 1987)	*P* value
Age, Median (IQR)	52 (35-66)	39 (28-55)	37 (29-48)	36 (27-46)	34 (25-46)	<0.001
Sex, % (No.)						
Female	51.1% (4678)	49.3% (1205)	49.2% (284)	48.6% (389)	51.7% (1028)	<0.001
Male	48.5% (4437)	50.3% (1227)	49.9% (288)	50.3% (403)	46.6% (926)	
Other or Prefer not to say	42 (0.5%)	10 (0.4%)	5 (0.9%)	1.1% (9)	1.7% (33)	
Race, % (No.)						
White, non-Hispanic	66.6% (6102)	63.1% (1540)	59.5% (343)	57.9% (464)	55.4% (1101)	<0.001
Black, non-Hispanic	10.2% (930)	10.1% (247)	10.6% (61)	11.1% (89)	12.0% (239)	
Asian, non-Hispanic	7.0% (642)	5.2% (126)	5.2% (30)	4.1% (33)	4.2% (83)	
Other, non-Hispanic	2.3% (211)	2.5% (62)	0.9% (5)	1.0% (8)	2.3% (46)	
Multiple race, non-Hispanic	2.0% (181)	1.8% (43)	1.4% (8)	1.5% (12)	2.4% (47)	
Hispanic or Latino, any race or races	11.9% (1091)	17.4% (424)	22.5% (130)	24.3% (195)	23.7% (471)	
In general, would you say that your health is						
Excellent or Very Good	46.3% (4243)	52.0% (1270)	56.5% (326)	59.2% (474)	48.9% (971)	<0.001
Good	35.5% (3253)	31.9% (779)	28.4% (164)	27.3% (219)	27.7% (550)	
Fair or Poor	18.1% (1661)	16.1% (393)	15.1% (87)	13.5% (108)	23.5% (466)	
Body Mass Index^a^,% (No.)						
Underweight	3.4% (291)	4.3% (95)	6.1% (30)	4.5% (30)	5.4% (92)	<0.001
Normal weight	34.0% (2955)	34.7% (776)	33.4% (164)	35.4% (236)	34.9% (597)	
Overweight	32.5% (2825)	31.2% (697)	30.1% (148)	33.2% (221)	26.4% (451)	
Obese	30.1% (2616)	29.8% (667)	30.4% (149)	26.9% (179)	33.3% (570)	
Diagnosed Medical Conditions, % (No.)						
Diabetes	15.2% (1393)	18.0% (439)	32.2% (186)	45.4% (364)	51.4% (1021)	<0.001
Hypertension	36.6% (3349)	31.2% (762)	41.1% (237)	54.3% (435)	59.9% (1190)	<0.001
Cardiovascular disease	10.3% (939)	11.6% (283)	25.5% (147)	41.2% (330)	47.3% (940)	<0.001
Chronic kidney disease	5.6% (517)	8.4% (205)	22.2% (128)	35.7% (286)	42.8% (850)	<0.001
COPD	7.0% (644)	9.4% (229)	24.3% (140)	37.1% (297)	44.6% (886)	<0.001
Asthma	14.6% (1332)	18.6% (454)	33.1% (191)	43.6% (349)	52.6% (1046)	<0.001
Anxiety	27.2% (2491)	31.2% (762)	47.3% (273)	58.1% (465)	70.4% (1398)	<0.001
Depression	29.4% (2694)	32.6% (796)	51.5% (297)	63.7% (510)	72.4% (1438)	<0.001
Obstructive sleep apnea	15.1% (1385)	18.6% (453)	35.5% (205)	50.1% (401)	57.0% (1132)	<0.001
Insomnia	23.7% (2170)	26.0% (636)	47.1% (272)	56.2% (450)	64.2% (1276)	<0.001
Cancer	10.9% (1002)	11.2% (273)	24.1% (139)	35.1% (281)	44.0% (875)	<0.001
Number of comorbidities, % (No.)						
0	31.5% (2886)	33.1% (808)	19.1% (110)	12.2% (98)	9.2% (182)	<0.001
1	20.7% (1898)	19.7% (482)	14.4% (83)	10.7% (86)	8.4% (166)	
2	17.1% (1567)	16.2% (396)	14.4% (83)	11.9% (95)	8.2% (163)	
3	12.0% (1101)	10.6% (258)	11.6% (67)	11.9% (95)	9.0% (179)	
≥4	18.6% (1705)	20.4% (498)	40.6% (234)	53.3% (427)	65.3% (1297)	
History of Infection^b^^,^ % (No.)						
Positive Test	None	63.7% (1555)	66.0% (381)	61.4% (492)	59.9% (1191)	0.02
Loss of Taste or Smell	None	50.1% (1224)	74.5% (430)	82.8% (663)	87.2% (1732)	<0.001
Clinical Diagnosis of COVID-19^c^	None	8.7% (213)	6.4% (37)	9.0% (72)	8.6% (171)	0.30
Infection Severity^b^, % (No.)						
Hospitalization	N/A	3.6% (89)	6.9% (40)	8.7% (70)	9.0% (179)	<0.001
Required oxygen	N/A	1.6% (40)	3.8% (22)	5.2% (42)	5.2% (104)	<0.001
Admitted to the ICU	N/A	1.4% (33)	2.8% (16)	3.9% (31)	4.3% (85)	<0.001

Table 1 Footnote: Statistical testing compares homogeneity between No PASC and PASC groups. PASC defined as any physical, cognitive or mood symptom. Chi-squared tests were used to compare proportions. Kruskal-wallis test was used to compare age.

a BMI was reported by 13,789 of the 14,964 respondents in this table.

b Statistical testing compares those with a history of infection (denominator = 5807).

c Without a positive test.

### 3.1 Prevalence of specific symptoms over 12 months in PASC survivors and unexposed controls

**Fig. 1:**
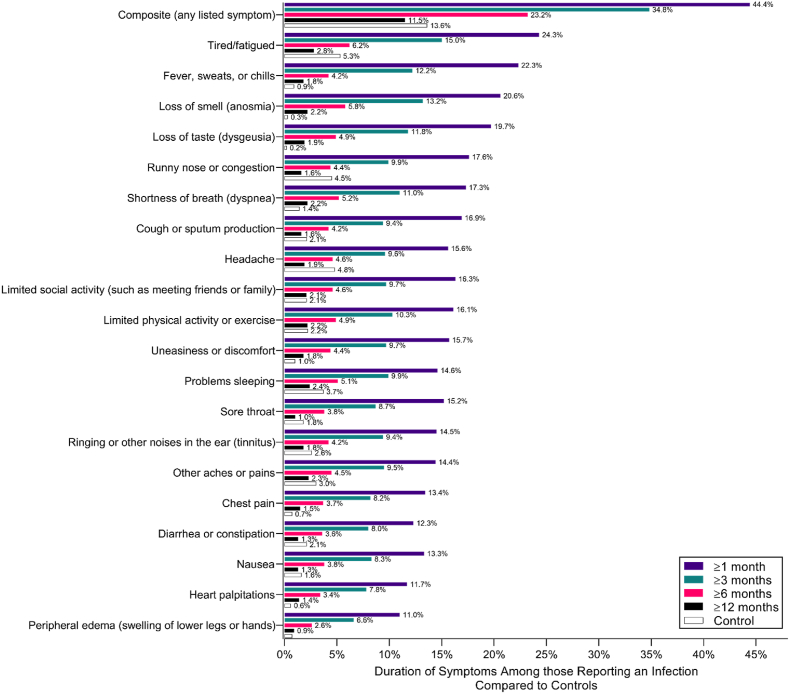
Duration of physical symptoms in cases vs controls over 12 months post-infection.

Overall, at 1-month post-infection, 44.4% of the sample reported at least one physical symptom (Fig. 1). The most common symptoms were fatigue (24.3%), fever, sweats or chills (22.3%), and loss of taste (19.7%) or smell (20.6%). Shortness of breath and cough were initially present in 17% post infection, and showed a steady decline over time. The prevalence of any self-reported physical symptom decreased to 34.8% at 3 months, 23.2% at 6 months and 11.5% at 12 months post-infection, and eventually to a similar rate to that of controls by 12 months post-infection (13.6%) (p > 0.05). By contrast, loss of taste and smell remained elevated (1.9-2.2%) compared to controls (0.2-0.3%) (*P* < 0.001).

**Fig. 2:**
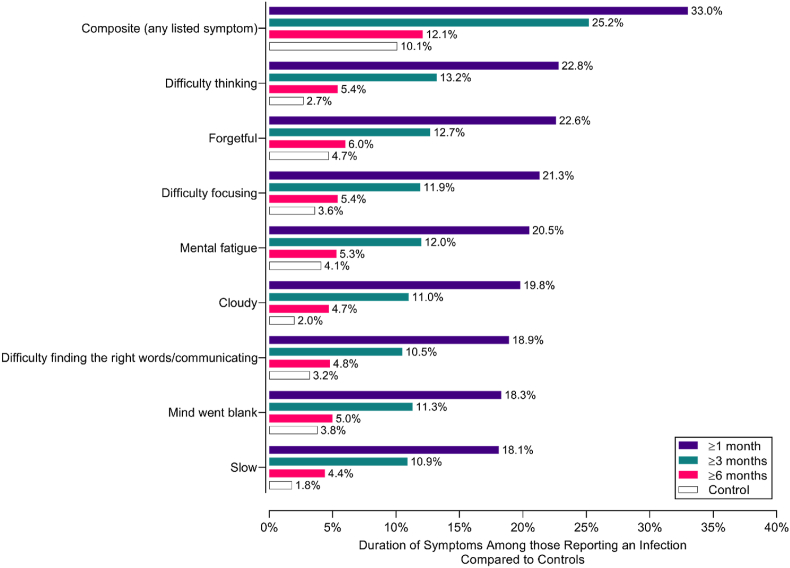
Duration of specific cognitive symptoms in cases vs controls over 6 months post-infection.

For cognitive symptoms, 33% of the sample reported at least one symptom at 1 month, declining to 25.2% at 3 months, 12.1% at 6 months, and to a similar level to controls thereafter (10.1% (p > 0.05); Fig. 2). A steady decline of specific symptoms was observed over time, but all remained elevated at 6 months relative to the controls, particularly “difficulty thinking”, “cloudy” and “slow” which remained double that of controls.

**Fig. 3:**
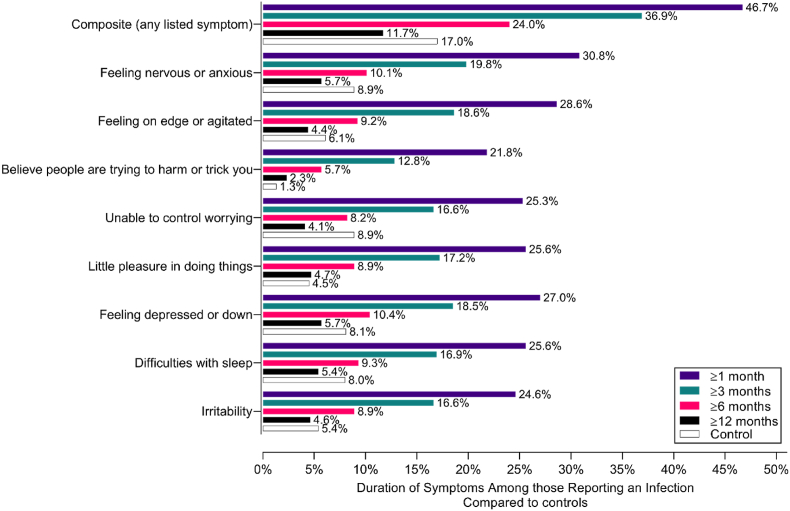
Duration of specific mental health symptoms in cases vs controls over 12 months post-infection.

Almost half (46.7%) of cases experienced at least one mental health symptom at 1-month post-infection (Fig. 3). This declined to 36.9% at 3 months, 24.0% at 6 months and 11.7% at 12 months post-infection. Interestingly, the prevalence of specific symptoms was higher in controls than cases at 12 months (17.0% vs. 11.7%). The prevalence of endorsement of either depression or anxiety using the PHQ-4 clinical cut-off for those cases and controls at baseline was 50.8% and 23.3%, respectively. Of those who reported PASC symptoms, the prevalence of individuals endorsing depression or anxiety symptoms remained elevated and higher over 12 months compared to individuals who reported a prior SARS-CoV-2 infection without endorsing any PASC symptoms (Fig. S1).

### 3.2 Cluster analyses

**Fig. 4:**
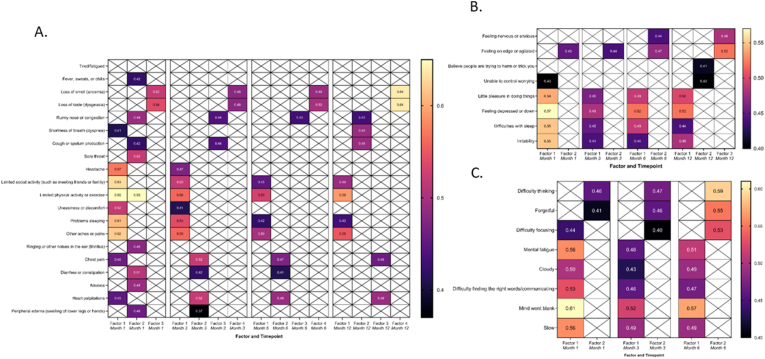
Rotated factor loadings for physical health (A), cognitive (B) and mental health (C) symptoms at 1 month, 3 months, 6 months, and 12 months post COVID-19 infection. Note: Numerical values represent final eigenvalues. Shading indicates the relative strength of the eigenvalue. Factors are arranged by column and time point of symptom persistence. Empty cells indicate that the symptom did not load onto that factor or loaded more strongly to another factor at that time point. Note data was collected out to 6 months for cognitive symptoms.

Three main physical clusters emerged at 1-month post-infection: Factor 1: Psychophysiological and autonomic dysfunction; Factor 2: Respiratory/gastrointestinal symptoms; and Factor 3: Loss of taste and smell (Fig. 4A). A fourth factor (runny nose/congestion and cough) emerged at 3-months post-infection, and persisted to 12-months.

Overall, loss of taste and smell (Factor 3) persisted over 12 months. Psychophysiological and autonomic dysfunction symptoms also persisted, although psychophysiological symptoms (limited social and physical activity, poor sleep, pain) were primarily retained in this cluster. In the Respiratory/gastrointestinal cluster, gastrointestinal and cardiovascular symptoms remained in the cluster at 3- and 6-months post-infection, and only heart palpitations and chest pain were present in the cluster at 12-months post-infection (Fig. 4). Respiratory symptoms separated into their own cluster at 3-12 months.

When comparing clusters to symptom prevalence figures, symptoms in the clusters present at 1, 3 and 6 months were generally much higher in PASC participants than controls. Some symptoms observed in the clusters at 12-months post-infection are also persistent in the general population, however, and therefore may not be specific to PASC (based on the duration and frequency of symptoms seen in controls; Fig. 1). For example, the prevalence of reporting runny nose and cough, and psychophysiological symptoms, was equivalent or higher in controls at 12-months (Fig. 1).

For adverse mental health symptoms (Fig. 4B), two distinct clusters emerged: Factor 1: Anxiety symptoms and Factor 2: Depressive symptoms. These clusters persisted out to 12 months, despite the prevalence of symptoms reducing over time. A “Paranoia” cluster emerged at 12 months post-infection. The prevalence of all symptoms was lower in PASC cases than controls at 12 months (Fig. 2).

Two cognitive factors emerged: Factor 1: Brain fog and Factor 2: Thinking and memory problems (Fig. 4C). These clusters persisted at 3- and 6-months post-infection. These symptoms were generally elevated compared to controls at 6 months, and were endorsed more frequently in PASC participants than controls (Fig. 2).

## 4 Discussion

In this cross-sectional survey study of nearly 15,000 demographically representative U.S. adults assessing PASC phenotypes among individuals with prior SARS-CoV-2 infection compared to never-infected individuals, we identified four distinct clusters of physical, cognitive, and adverse mental health symptoms. Approximately 1 in 2 individuals reported experiencing at least one symptom for each cluster at 1-month post infection, which progressively declined between 6- and 12-months post-infection to general population levels for most symptoms. Results from the cluster analysis revealed a persistence in loss of taste and smell over 12-months, with specific psychophysiological and gastrointestinal/respiratory/cardiovascular symptoms also persisting at 12 months post-infection. Cognitive clusters persisted out to 6 months post-infection, and mental health clusters persisted to 12 months post-infection. Individuals with PASC who had at least one symptom from a cluster were more likely to experience depression or anxiety compared to controls.

The physical symptom phenotypes emerging from our cohort included 3 primary clusters: loss of taste and smell, psychophysiological/autonomic dysfunction and respiratory/cardiac/gastrointestinal symptoms, which persisted over 12 months. Of note, loss of taste and smell was reported by ∼20% of the sample post-infection, and this cluster persisted for 12 months, consistent with previous longitudinal (Boscolo-Rizzo et al., 2022; Jafar et al., 2021) and uncontrolled studies examining PASC phenotypes (Pinto et al., 2022). Olfactory dysfunction caused by SARS-CoV-2 typically resolves in most people after a few weeks, however previous studies estimate 25-30% have long-term and likely permanent deficits 1-year post-infection (Doty, 2022). Some previous reports included outpatient cohorts and used objective measures of olfactory dysfunction, which may explain the higher figures compared to the current study. Potential pathophysiological mechanisms include reversible inflammation within the olfactory cleft, damage to the olfactory mucosa and longer-term injury to the peripheral and central olfactory structures or circuits, which may have implications for neurological and psychiatric sequelae in COVID-19 cases (Doty, 2022).

In addition to olfactory dysfunction, psychophysiological/autonomic dysfunction was also reported, particularly at ≥6 months timepoint. These symptoms may reflect fatigue, generalized aches and pains, chest pain, poor sleep, palpitations or breathlessness - reflecting lingering inflammation, or dysregulation of the autonomic nervous system. Studies show that individuals with Long COVID may experience dysautonomia, or other autonomic symptoms, including postural tachycardia, gastrointestinal dysregulation, and exertional intolerance, which is also consistent with other post-viral syndromes and myalgic encephalomyelitis/chronic fatigue syndrome. This clustering of psychophysiological symptoms represents a target for rehabilitation and functional interventions.

Regarding the prevalence of specific symptoms, individual constitutional, psychophysiological and cardiorespiratory symptoms were common (each 10-20% at one month), consistent with previous reports (Callejón-Leblic et al., 2022). Cases reported an elevated prevalence of these symptoms relative to controls during the first 6 months after infection. Respiratory and physical limitations may result from the initial impacts of infection on pulmonary, cardiac and muscle function. However, both psychophysiological and cardiorespiratory symptoms have also been observed in non-hospitalized populations, reflecting that these symptoms may evolve independently of acute disease severity. Symptoms within the physical clusters may change over time, which can reflect both the changes or reduction in symptoms across time. This may also be attributed to changes in the prevalence of SARS-CoV-2 strains across Waves 1-3. For example, PASC was more common in the pre-Omicron variant era; brain fog was more prevalent after infection with the delta variant than with the alpha variant (Canas et al., 2023). Our survey data was collected post Omicron, so participants could have been infected in any of the previous waves.

We observed a clustering of brain fog and impaired thinking/memory symptoms, which persisted throughout the 6-month reporting period. Previous longitudinal studies in general community samples have reported a prevalence of cognitive symptoms post SARS-CoV-2 infection of ∼36% (Liu et al., 2023), with 1 in 5 experiencing “brain fog” >3 months post-infection (Ceban et al., 2022); these estimates are in line with our findings of 25% prevalence of any cognitive symptom at 3 months post-infection. A meta-analysis of 2 million symptomatic COVID-19 patients reported that 2.2% experience chronic cognitive symptoms 3 months post-infection (GBoDLC, 2022). Persistence of cognitive deficits has been observed 2 years post-infection in patients with a recorded diagnosis of COVID-19 compared to patients with another respiratory infection (Taquet et al., 2022). These protracted cognitive symptoms are reminiscent of other post-infective fatigue syndromes and myalgic encephalomyelitis/chronic fatigue syndrome (Komaroff and Lipkin, 2021). While the pathogenesis of these cognitive symptoms is still unclear, one explanation is the neurological sequalae of SARS-CoV-2 infection, including systemic inflammation with associated heightened cytokine production, and increased blood brain barrier permeability leading to neuroinflammation or direct viral invasion of the brain (Reiss et al., 2023; Zhao et al., 2023).

Our study identified two distinct clusters of depressive and anxiety symptoms, which persisted across the 12-month reporting period. These clusters emerged at 1-month post-infection, and were sustained at 3 months; however, the prevalence of specific mental health symptoms was similar to controls by 6 months (17% vs 24%). Mood disorders typically show a transient profile, with a risk horizon of less than 2 months, and equivalent incidence at around 15 months (Taquet et al., 2022). Our data reporting *symptoms* over time are likely to predate a diagnosis by weeks or months. In other studies, the estimated incidence of a new-onset neurological or psychiatric diagnosis was 11.5% in the first 6 months after infection, which doubled among patients with an ICU admission. Mental health symptoms are common after ICU admission for any cause, and may not be specific to SARS-CoV-2 infection (Harrison and Taquet, 2023), although reported rates are higher than those observed after influenza (Taquet et al., 2021). Studies suggest that inflammatory processes may contribute to acute mood symptoms, particularly anxiety (Michopoulos et al., 2017). Pro-inflammatory cytokines are implicated in the pathophysiology of mood disorders, but other biopsychosocial factors may play a critical role in maintenance of these disorders. Our cohort predominantly included a non-clinical, non-hospitalized population, and yet the prevalence of mental health symptoms remained elevated relative to controls for 6 months after infection, suggesting that altered mental health is not just restricted to those with severe acute disease. Other studies have reported approximately 10% of patients with milder infection managed in the community reported adverse mental health symptoms 13 weeks post-infection (Van den Borst et al., 2020).

Many symptoms observed in the clusters at 12-months post-infection, particularly adverse mental health and cognitive symptoms, were also prevalent in the general population control group, suggesting they may reflect broader community-level prevalence of these symptoms, rather than PASC specifically. High rates of adverse mental health and cognitive symptoms were reported in controls at 6 and 12 months. This is consistent with global increase in the prevalence of anxiety, depression and sleep disturbance during the early stages of the pandemic (Varma et al., 2021; Wu et al., 2021), as observed after other epidemics and natural disasters (Beaglehole et al., 2018; Cheng et al., 2004). Explanatory factors include concern about exposure to the virus, changes to living and working arrangements due to stay-at-home orders (Brooks et al., 2020), and social isolation. It should be noted that over 90% of those who had SARS-CoV-2 infection in the current study were managed in the community, limiting generalizability to those with severe illness. Notably, ongoing cognitive and psychiatric symptoms are common after any critical illness (Pandharipande et al., 2013); and a United Kingdom study using primary care records from over 32,500 COVID-19 cases found no increased incidence of new-onset anxiety or depression after infection was observed compared to other respiratory infections (Clift et al., 2022).

This study examined prevalence and clusters of PASC symptomology in US adults using non-probabilistic sampling to approximate population estimates for age, sex, race and ethnicity. The large sample size, comprehensive characterization of symptom clusters from 1 to 12 months post infection and comparison with a control population with no prior history of infection enhances understanding of PASC symptoms and how they evolve over time. Some limitations should be noted. First, ascertainment of SARS-CoV-2 infection and the presence of mental health and cognitive symptoms were by self-report which may have resulted in misclassification and/or bias. In particular, cognitive outcomes were symptoms based self-report and not determined by objective assessment, due to the large-scale nature of this cohort. Similarly, mood symptoms were self-reported and not clinician diagnosed. Second, the study was cross-sectional and participants were not observed longitudinally, which may result in recall bias. Third, it is difficult to determine cause and effect between physical, adverse mental health and cognitive symptoms. PASC cognitive impairment can occur regardless of mental health symptoms. Indeed, data from electronic health records indicated that mental health symptoms returned to normal within 2 months, whereas cognitive symptoms increase for at least 2 years (Harrison and Taquet, 2023). Further, the relationship between the presence of olfactory symptoms and persisting physical, cognitive or mental health symptoms warrants further investigation (Jegatheeswaran et al., 2023).

Post-acute sequelae of COVID-19 represent a significant burden on healthcare systems globally, with a diverse array of persistent symptoms affecting various organ systems. These key symptom clusters included loss of taste and smell, psychophysiological, cardiovascular and respiratory symptoms, and brain fog, although the majority of symptoms from this general population study resolved within 12 months. Understanding the underlying pathophysiological mechanisms for these symptom clusters, particularly those associated with olfactory and neurological sequalae, is essential for guiding clinical management strategies, rehabilitation programs, and public health interventions aimed at mitigating the long-term impact of COVID-19 on individuals and society as a whole.

## CRediT authorship contribution statement

**Melinda L. Jackson:** Conceptualization, Methodology, Writing – original draft. **Matthew D. Weaver:** Conceptualization, Data curation, Formal analysis, Funding acquisition, Investigation, Methodology, Project administration, Supervision, Visualization, Writing – review & editing. **Prerna Varma:** Conceptualization, Data curation, Methodology, Visualization, Writing – review & editing. **Mark É. Czeisler:** Data curation, Funding acquisition, Investigation, Methodology, Project administration, Writing – review & editing. **Lauren A. Booker:** Methodology, Writing – review & editing. **Christine F. McDonald:** Investigation, Methodology, Writing – review & editing. **Rebecca Robbins:** Funding acquisition, Methodology, Writing – review & editing. **Anna Ridgers:** Methodology, Writing – review & editing. **Rashon Lane:** Funding acquisition, Investigation, Methodology, Project administration, Writing – review & editing. **Shantha M.W. Rajaratnam:** Funding acquisition, Investigation, Methodology, Project administration, Resources, Writing – review & editing. **Charles A. Czeisler:** Funding acquisition, Investigation, Methodology, Project administration, Resources, Writing – review & editing. **Stuart F. Quan:** Conceptualization, Funding acquisition, Investigation, Methodology, Writing – review & editing. **Mark E. Howard:** Conceptualization, Funding acquisition, Investigation, Methodology, Resources, Writing – review & editing.

## Disclosures

The COPE Initiative was supported by the United States Centers for Disease Control and Prevention. The salaries of MDW and RR were supported, in part, by National Heart, Lung, and Blood Institute (NHLBI) R56 HL151637 and NHLBI K01 HL150339, respectively. M.D.W. reports institutional support from the United States Centers for Disease Control and Prevention, National Institutes of Occupational Safety and Health, and Delta Airlines as well as consulting fees from the Fred Hutchinson Cancer Center and the University of Pittsburgh. M.É.C. was supported by an Australian-American Fulbright Fellowship, with funding from The Kinghorn Foundation, research grants or gifts to Monash University from WHOOP, Inc., Hopelab, Inc., CDC Foundation, and the United States Centers for Disease Control and Prevention, as well as consultancy fees from Nychthemeron, LLC. R.R. reports personal fees from Hilton Hotels, LLC, Oura Ring Ltd, byNacht GmpH. R.R. serves on the Medical Advisory Boards for Oura Ring, Equinox Fitness Clubs, and Somnum Pharmaceuticals. S.F.Q. reports service as a consultant for Teledoc, Bryte Foundation, Jazz Pharmaceuticals, Apnimed, and Whispersom. S.M.W.R. reported receiving grants and personal fees from Cooperative Research Center for Alertness, Safety, and Productivity, receiving grants and institutional consultancy fees from Teva Pharma Australia and institutional consultancy fees from Vanda Pharmaceuticals, Circadian Therapeutics, BHP Billiton, and Herbert Smith Freehills. CAC reported receiving grants and personal fees from Teva Pharma Australia, receiving grants from the National Institute of Occupational Safety and Health R01-OH-011773, personal fees from and equity interest in Vanda Pharmaceuticals Inc, educational and research support from Philips Respironics Inc, an endowed professorship provided to Harvard Medical School from Cephalon, Inc, an institutional gift from Alexandra Drane, and a patent on Actiwatch-2 and Actiwatch-Spectrum devices with royalties paid from Philips Respironics Inc. CAC's interests were reviewed and managed by Brigham and Women's Hospital and Partners HealthCare in accordance with their conflict of interest policies. CAC also served as a voluntary board member for the Institute for Experimental Psychiatry Research Foundation, Inc. No other conflicts of interest are reported.

## Funding information

Data collection was supported by the United States Centers for Disease Control and Prevention.

## Declaration of competing interest

The authors declare the following financial interests/personal relationships which may be considered as potential competing interests:COPE Initiative reports financial support was provided by Centers for Disease Control and Prevention. Matthew Weaver reports a relationship with National Heart Lung and Blood Institute that includes: funding grants. Matthew Weaver reports a relationship with National Institute of Occupational Safety and Health that includes: funding grants. Matthew Weaver reports a relationship with Fred Hutchinson Cancer Center that includes: consulting or advisory. Matthew Weaver reports a relationship with University of Pittsburgh that includes: consulting or advisory. Mark Czeisler reports a relationship with The Kinghorn Foundation that includes: funding grants. Mark Czeisler reports a relationship with HopeLab Foundation that includes: funding grants. Mark Czeisler reports a relationship with National Foundation for the Centers for Disease Control and Prevention Inc that includes: funding grants. Rebecca Robbins reports a relationship with Hilton Hotel that includes: consulting or advisory. Rebecca Robbins reports a relationship with Ōura Health Oy that includes: board membership and consulting or advisory. Rebecca Robbins reports a relationship with Equinox that includes: board membership. Rebecca Robbins reports a relationship with Somnum that includes: consulting or advisory. Stuart Quan reports a relationship with Jazz Pharmaceuticals Inc that includes: consulting or advisory. Stuart Quan reports a relationship with Apnimed Inc that includes: consulting or advisory. Shantha Rajaratnam reports a relationship with Cooperative Research Centers Program that includes: funding grants. Shantha Rajaratnam reports a relationship with Teva Pharma Australia Pty Ltd that includes: consulting or advisory and funding grants. Shantha Rajaratnam reports a relationship with Vanda Pharmaceuticals Inc. That includes: consulting or advisory. Shantha Rajaratnam reports a relationship with Circadian Therapeutics Limited that includes: consulting or advisory. Shantha Rajaratnam reports a relationship with BHP Billiton Ltd that includes: consulting or advisory. Shantha Rajaratnam reports a relationship with Herbert Smith Freehills Kramer LLP that includes: consulting or advisory. Charles A. Czeisler reports a relationship with Teva Pharma Australia Pty Ltd that includes: consulting or advisory and funding grants. Charles A. Czeisler reports a relationship with National Institute of Occupational Safety and Health that includes: funding grants. Charles A. Czeisler reports a relationship with Vanda Pharmaceuticals Inc that includes: consulting or advisory and equity or stocks. Charles A. Czeisler reports a relationship with Philips Respironics Ltd that includes: funding grants and speaking and lecture fees. Charles A. Czeisler reports a relationship with Teva Pharmaceuticals USA Inc that includes: funding grants. Charles A. Czeisler reports a relationship with Institute for Experimental Psychiatry Research Foundation that includes: board membership. Has patent pending to. If there are other authors, they declare that they have no known competing financial interests or personal relationships that could have appeared to influence the work reported in this paper.

## Data Availability

All relevant data supporting the findings are available from the corresponding author upon request. Reuse is permitted only following a written agreement from the corresponding author and Institution
