# Supplementary material for: Presentation and temporal nature of postacute sequelae of SARS-CoV-2 infection in a US national cohort
Source: Brain Behav Immun Health. 2026 Feb 27;53:101205. doi: 10.1016/j.bbih.2026.101205 (PMC12969318; doi:10.1016/j.bbih.2026.101205)
Supplement: Multimedia component 1 [file mmc1.docx]

**Table S1.** Demographics of the overall sample

|  | **Overall**  **100% (n=14,964)** |
| --- | --- |
| Age |  |
| 18-24 | 15.1% (2,267) |
| 25-44 | 33.0% (4,942) |
| 45-64 | 32.7% (4,885) |
| ≥65 | 19.2% (2,870) |
| Race |  |
| White, non-Hispanic | 63.8% (9,550) |
| Black, non-Hispanic | 10.5% (1,566) |
| Asian, non-Hispanic | 6.1% (914) |
| Other, non-Hispanic | 2.2% (332) |
| Multiple race, non-Hispanic | 1.9% (291) |
| Hispanic or Latino, any race or races | 15.4% (2,311) |
| Sex at Birth |  |
| Female | 50.7% (7,584) |
| Male | 48.7% (7,281) |
| Other | 1.5% (227) |
| Prefer Not to Say | 0.1% (21) |
| Education |  |
| High school diploma or less | 27.4% (4,092) |
| Some college or college | 54.9% (8,212) |
| Master’s, professional, or doctoral degree | 17.8% (2,660) |
| Employment |  |
| Full-time | 40.1% (6,000) |
| Part-time | 10.2% (1,533) |
| Unemployed | 16.0% (2,390) |
| Retired | 23.0% (3,451) |
| Other | 10.6% (1,590) |
| Total Household Income in 2021 |  |
| Less than $25,000 | 20.5% (3,068) |
| $25,000-$49,999 | 22.7% (3,399) |
| $50,000-$99,999 | 27.9% (4,169) |
| $100,000-$199,999 | 19.7% (2,950) |
| $200,000 or more | 4.9% (731) |
| Prefer not to say | 4.3% (647) |

**Table S2.** Timeline of data collection.

| **Timeline of Data Collection** | **Surveys Collected** |
| --- | --- |
| March 10-30, 2022 | 4,997 |
| April 4 – May 1, 2022 | 4,983 |
| May 4 – June 2, 2022 | 4,984 |
| **Total** | 14,964 |

**Supplement S3** – PASC Symptom items

In the **last two weeks**, have you experienced any of the following symptoms *(select all that apply)*

**Physical**

Fever, sweats, or chills

Tired/fatigued

Loss of smell (anosmia)

Loss of taste (dysgeusia)

Runny nose or congestion

Sore throat

Ringing or other noises in the ear (tinnitus)

Shortness of breath (dyspnea)

Cough or sputum production

Nausea

Uneasiness or discomfort

Diarrhea or constipation

Chest pain

Heart palpitations

Peripheral edema (swelling of lower legs or hands)

Headache

Excessive sleepiness

Problems sleeping

Limited physical activity or exercise

Limited social activity (such as meeting friends or family)

Other aches or pains

Other

None of the above

**Cognitive**

Forgetful

Difficulty thinking

Difficulty focusing

Cloudy

Difficulty finding the right words/communicating

Mental fatigue

Slow

Mind went blank

Other

None of the above

**Mental Health**

Feeling nervous or anxious

Feeling on the edge or agitated

Believe people are trying to harm or trick you, even if there's no evidence

Unable to control worrying

Little pleasure in doing things

Feeling depressed or down

Difficulties with sleep (e.g. trouble falling/staying asleep, restlessness, nightmares)

Irritability

Avoiding places, people or situations that remind of COVID-19 disease

None of the above


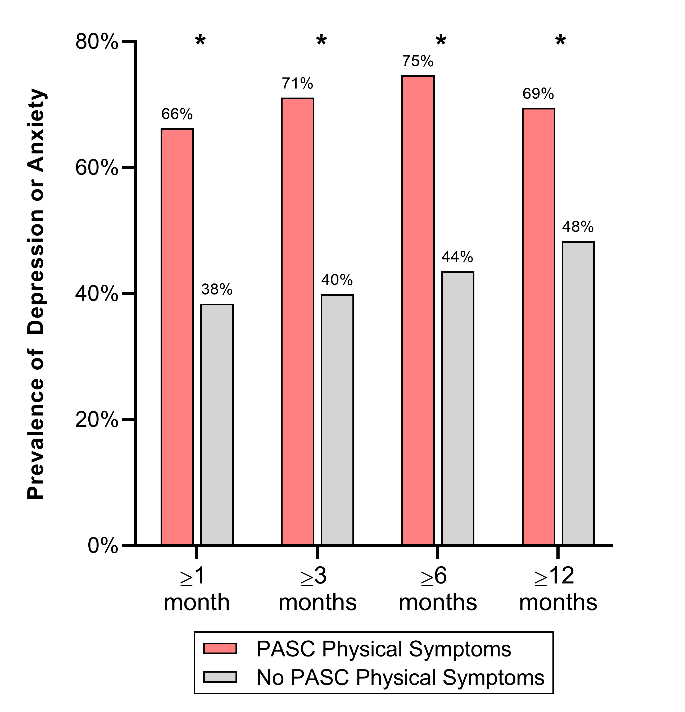
**A**

**B C**


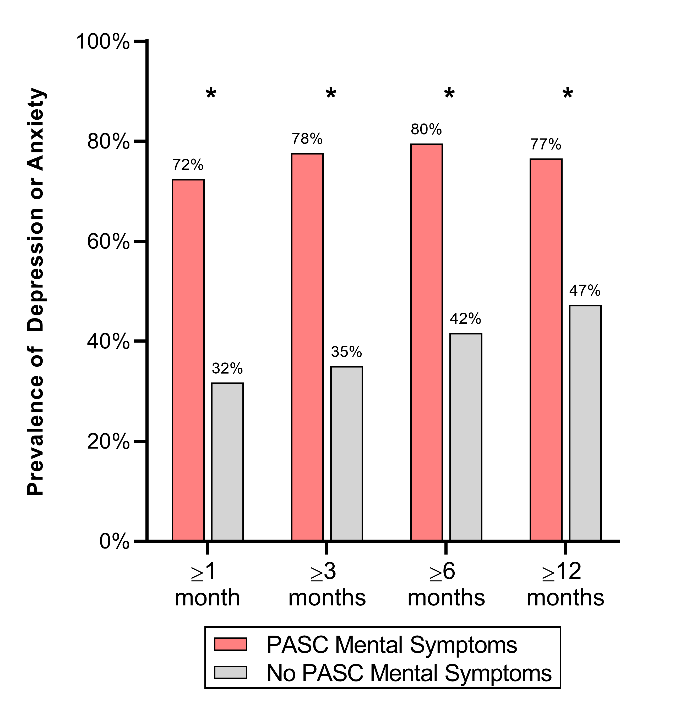

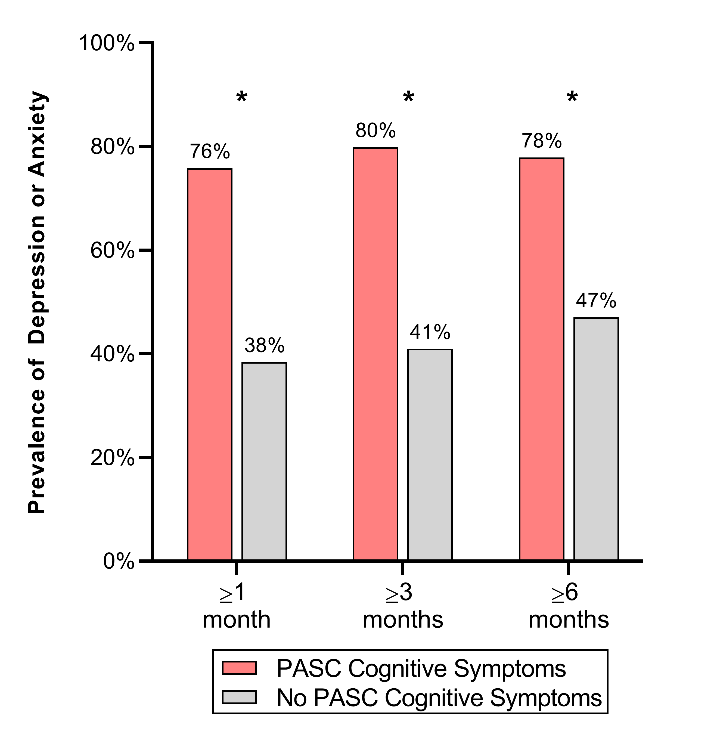


**Figure S1:** Prevalence of significant depression or anxiety symptoms, using clinical cut-offs on the PHQ-4, for respondents who endorsed one or more physical (A), cognitive (B) and mental health (C) symptoms compared to respondents who reported a prior SARS-CoV-2 infection but no PASC symptoms at each time point. ***** Indicates significant difference in proportion using Chi-Squared tests for proportions.
